# Changes in serum prolactin level during intracytoplasmic sperm injection, and effect on clinical pregnancy rate: a prospective observational study

**DOI:** 10.1186/s12884-018-1783-4

**Published:** 2018-05-09

**Authors:** Ahmed Kamel, Ayman A. Halim, Mohamed Shehata, Salwa AlFarra, Yahia El-faissal, Wafaa Ramadan, Ahmed M. Hussein

**Affiliations:** 1 Obstetrics and Gynecology Department, Cairo University, P.O. Box: 11562, KasrAlainy St., Garden City, Cairo, Egypt; 2 Clinical and Chemical Pathology Department, Cairo University, Cairo, Egypt

**Keywords:** Embryo quality, Intracytoplasmic sperm injection, Hyperprolactinemia, Prolactin

## Abstract

**Background:**

Transient hyperprolactinemia was proven to adversely affect the outcome of IVF. We aimed to identify changes in serum prolactin levels in patients undergoing ICSI, and to evaluate the effect of these changes on the clinical pregnancy rate.

**Methods:**

A prospective observational study included 90 patients scheduled for ICSI cycles. In each case 4 serum samples were collected during the cycle (midluteal, before ovum pick up procedure (OPU), 2 h after OPU, and before embryo transfer). Serum prolactin level was determined by immunoassay each time.

**Results:**

The sample collected 2 h after OPU had a mean difference of 25.8 ± 2.8 ng/ml compared to the basal serum prolactin (*p* < 0.01). In comparison to other samples, this highlighted a significant hyperprolactinemia occurring after OPU, and resolving before embryo transfer. No statistically significant difference between the different serum prolactin samples amongst the pregnant and non pregnant patients. There was a significant positive pearson correlation between the prolactin levels before OPU, and the presence of higher quality embryos (*r* = 0.274, *p* = 0.019).

**Conclusion:**

In normoprolactinemic women transient hyperprolactinemia is identified in patients undergoing ICSI, and it doesn’t affect the clinical pregnancy rates. A positive correlation was identified between higher quality embryos, and serum prolactin level before OPU.

**Trial registration:**

ClinicalTrials.gov Identifier: NCT02292953, First received: November 10, 2014.

## Background

Prolactin (PRL) is a 198 amino acid polypeptide hormone secreted in a pulsatile manner from the anterior pituitary, and is synthesized by lactotroph cells. It weighs 23 kDa, and its secretion is affected by many factors which include stress, sleep, pregnancy, ingesting food, and chest wall stimulation (trauma, or pain). Prolactin has a primary role in the regulation of reproductive functions. While the elevation of its level beyond normal (hyperprolactinemia) can be either physiological, pathological, or idiopathic; its clinical manifestations can vary from severe to none [1].

Prolactin receptors have been found on endometrial cells. The secretion of endometrial prolactin helps in maintaining endometrial receptivity, and has been shown to provide an optimal environment for the implanting blastocyst transferred during in-vitro fertilization (IVF) cycles. A high enough prolactin level can inhibit the proliferation of luteinizing granulosa cells, and can also interfere with corpus luteum function resulting in luteal phase defect, as well as abnormal implantation, and embryo development [2]. Different studies have proven the existence of a state of hyperprolactinemia during IVF/Intracytoplasmic sperm injection (ICSI) cycles [3, 4].

Transient hyperprolactinemia has been proven to adversely affect the outcome of IVF, and studies have concluded that patients with normal serum prolactin levels had higher fertilization and cleavage rates [5–7]. While other researchers found the effect of increased serum prolactin during IVF to be insignificant [8–10], subsequent studies [11–14] found a positive effect of transient hyperprolactinemia on the outcome of ovarian simulation in IVF.

The aim of this study was to identify changes in serum prolactin levels in patients undergoing ICSI, and to evaluate the effect of these changes on the clinical pregnancy rate.

## Methods

### Experimental design & sample size

An internal review board “The Scientific & Ethics committee” of the OBGYN department, Cairo University approved this study protocol on the fifth of October 2014. One hundred two normo-prolactinemic patients were recruited in the study while attending the assisted reproduction clinic in Kasr Alainy, Cairo university hospital. All enrolled subjects were already scheduled for ICSI. Each participant was subjected to investigations which included follicle stimulating hormone (FSH), Luteinizing hormone (LH), thyroid stimulating hormone (TSH), prolactin, and semen analysis for the husband.

The sample size was calculated using an online calculator [15] where the measured outcome was the difference of serum prolactin levels between samples. The confidence level was set at 95%, and the margin of error at 10.4%, in a 50% response distribution, giving us 90 patients in a population more than 20,000.

Inclusion Criteria for the study included an age range between 20 and 38 years, women with either 1ry or 2ry infertility scheduled for ICSI. Exclusion from the study was due to hyperprolactinemia (high basal serum prolactin), uterine anomalies, repeated ICSI failure (3 or more failed trials), embryo transfer beyond day 3, frozen –thawed cycles, or had any contraindications for pregnancy (serious uncontrolled medical diseases such as diabetes, heart disease, hypertension, or ovarian tumors (cancer of any location). Patients were also excluded if they were on any medication that is known to alter prolactin levels e.g. antipsychotics and risperidone, or had a history of thyroid dysfunction (e.g. low TSH), or had any medical disorders that affect serum prolactin e.g. acromegaly and chronic renal failure.

After applying inclusion and exclusion criteria, the patients were scheduled to start ICSI cycle at Kasr Alainy assisted reproduction unit. Patient counseling and informed consent was taken regarding all blood samples that will be drawn, and permission for publishing this data was granted.

The first baseline serum prolactin level was recorded in the mid-luteal phase of the cycle preceding ICSI. The second blood sample was taken in operating room just before induction of anesthesia for OPU. A Third blood sample was taken two hours after aspiration of the last follicle in recovery room post-operatively. A Fourth and final blood sample was taken before embryo transfer. To avoid fluctuations in serum prolactin levels, all OPU and embryo transfers (ET) was scheduled at 9:00 am after fasting for 8 h. The blood samples were immediately sent to central laboratory of Kasr Alainy Cairo university hospital after retrieval. Serum prolactin was analyzed each time though immunoassay by ADVIA Centaur Immunoassay System (CP Immunoassay System ®, SIEMENS AG, Munich, Germany).

### Intracytoplasmic sperm injection

The standard long gonadotrophin-releasing hormone (GnRH) agonist protocol was used for all patients where 0.1 mg of Triptorelin daily S.C injection *(Decapeptyl ®FERRING PHARMACEUTICALS, Switzerland)* was applied from the mid-luteal phase onward till the day of human chorionic gonadotropin (hCG) injection. When the serum estradiol (E2) level was < 50 pg/ml, gonadotropins in the form of human menopausal gonadotropin (HMG) (Merional ®, IBSA, InstitutBiochimique SA, Lugano, Switzerland) were given IM from the 3rd day of menstruation. The starting dose of HMG was set from 150 to 300 IU according to the patient’s age, basal FSH level, antral follicular count (AFC), and body mass index (BMI).

The stimulation was monitored by transvaginal ultrasonography, and serial E2 measurements starting from the seventh day of the cycle, and the HMG dose was adjusted individually according to follicular response. After the development of three or more follicles ≥18 mm, 10,000 unit of hCG (Choriomon, IBSA, Institut Biochimique SA) was given IM, and trans-vaginal ultrasound guided ovum pick up procedure (OPU) was scheduled 36 h later. ICSI was performed as described by *Mansour* et al. [16].

Progesterone 100 mg IM daily injections (Prontogest *® IBSA, InstitutBiochimique SA, Lugano, Switzerland*) were given as luteal support starting from the day of ET, and continued for 16 days. Pregnancy was recorded when β-HCG > 10 IU on day 14 after ET, and a second higher value 48 h later. This was followed by ultrasonography for confirmation of fetal pole and cardiac activity at 7 weeks of gestation.

Descriptive data such as age, cause and type of infertility, the number of oocytes retrieved at OPU, and embryo grading were recorded as well as the result of every serum prolactin sample.

### Statistical analysis

Continuous data were expressed as mean ± SD (95% confidence interval [CI]), and were compared using Student t test or Mann-Whitney U test as appropriate. General linear model for repeated measures was used to evaluate the mean pair wise comparison between different prolactin levels where Bonferroni adjustment was used in the estimated marginal means. Correlation between various variables was done using Pearson moment correlation equation. Statistical analysis was performed using the Statistical Package for the Social Sciences program, v20.0 (SPSS Inc., Chicago, IL, USA). A two-tailed *P* value < 0.05 was considered statistically significant.

## Results

The total number of participants in our study was 102 patients. During the course of our study twelve cases were excluded as the embryo transfer procedure was cancelled. Three of cancelled cases were identified as high risk for ovarian hyperstimulation, and had their embryos frozen for transfer in another cycle. In four cases, no oocytes were retrieved, and in another four cases only germinal vesicles (GV) were retrieved, and not injected. There was a single case of failed fertilization. Recruitment then continued till 90 patients were enrolled.

### Descriptive data

33 patients out of 90 got pregnant (36%), only 27 of which were clinical pregnancies, giving us 30% clinical pregnancy rate. The implantation rate was 19.3%, and the live birth rate was 23.3%. Primary infertility represented 61.1% of cases, tubal factor comprised 40% of the cases, unexplained infertility was 26.6%, male factor was 20%, and unovulatory polycystic ovarian syndrome (PCOS) was 13.4%. Endometriosis was identified in 17% of cases with tubal factor.

Descriptive statistics are illustrated in Table 1, where the ovarian reserve of the participants appears normal as denoted by the AFC, serum FSH and LH. The mean BMI was 29.1 ± 1.5 kg/m^2^, and there was no statistical significance in the BMI between the patients that got pregnant and those that didn’t (*p* = 0.846).

**Table 1:** Descriptive statistics showing different hormone levels & factors affecting ICSI outcome

	Minimum	Maximum	Mean ± SD
Age (years)	20	38	29.6 ± 5.4 (28.7–30.6)
AFC (follicles)	2	32	13 ± 6.3 (11.7–14.4)
FSH (IU/mL)	1.89	16.4	6 ± 2.9 (6–7.2)
LH (IU/mL)	1.1	13.9	5.2 ± 2.8 (4.7–5.8)
TSH (mIU/L)	0.7	4.1	2.01 ± 0.84 (1.83–2.18)
BMI (kg/m^2^)	25.1	33	29.1 ± 1.5 (28.8–29.4)
Basal PRL level (ng/ml)	1.1	28	13.7 ± 6.6 (12.3–15.1)
PRL before OPU (ng/ml)	0.4	46.2	16.8 ± 11. 6 (14.4–19.2)
PRL after OPU (ng/ml)	0.5	93.2	39.5 ± 25.4 (34.2–44.8)
PRL before ET (ng/ml)	0.1	73.6	11. 5 ± 10 (9.4–13.6)
Number of oocytes retrieved in OPU	2	44	17.3 ± 10.7 (15–19.5)
Number of metaphase II oocytes	1	13	5.7 ± 2.9 (5.1–6.4)
Fertilization rate (%)	40	100	81.5 ± 16 (78.2–85)
Number of embryos transferred	1	5	3.2 ± 1.1 (3–3.4)
Number of high quality embryos	0	5	2.9 ± 1.3 (2.6–3.1)
Number of low quality embryos	0	2	1.1 ± 0.3 (1–1.2)

Values are given in given in numbers, & mean ± SD (95% CI)

*SD* standard deviation

### Transient hyperprolactinemia

The mean level of prolactin before OPU was within the normal range, while the level after OPU was elevated beyond normal reaching a maximum of 93.2 ng/ml. However the mean levels of PRL before embryo transfer did fall back to normal levels and a maximum value of 73.6 ng/ml was recorded.

The difference between the mean basal prolactin levels and the other serum samples collected is shown in Table 2. The sample collected 2 h after OPU had a statistically significant mean difference of 25.8 ± 2.8 ng/ml in comparison to the basal level of serum prolactin (*p* < 0.01). This helps highlight a significant hyperprolactinemia occurring after OPU, and resolving before embryo transfer. There was no statistical difference between the other samples, and the basal prolactin level as shown in Table 2.

**Table 2:** The mean difference between different levels of serum prolactin compared to the basal level

	Mean Prolactin level ng/ml	Mean difference from basal PRL level ng/ml	95% confidence interval		*p*-value
			Lower bound	Upper bound	
PRL before OPU	16.8 ± 11.6	3.1 ± 1.4	0.556	6.816	0.146
PRL after OPU	39.5 ± 25.4	25.8 ± 2.8	18.205	33.391	< 0.01^*^
PRL before ET	11.5 ± 10	2.2 ± 1.1	0.852	5.29	0.326

Values are given as mean ± standard error (95% CI)

^*^Statistically significant result *p* < 0.05

(F = 406.9, *p* < 0.001)

Table 3 compares the mean prolactin levels between patients who had a clinical pregnancy to those who didn’t. There was also no statistically significant difference between the different prolactin samples amongst the two groups even though there was a statistically significant rise in serum prolactin in the sample taken 2 h after OPU.

**Table 3:** Comparison between the clinical parameters, and mean levels of serum prolactin in the pregnant and non pregnant groups

	Pregnant	Non pregnant	*P*-value
Age (years)	29.2 ± 6 (26.9–31.6)	29.8 ± 5.2 (28.7–31.1)	0.644
AFC (follicles)	15.1 ± 7.2 (12.3–17.9)	12.1 ± 5.7 (10.7–13.5)	0.101
FSH (IU/mL)	7 ± 3.4 (5.7–8.3)	6.4 ± 2.6 (5.8–7.1)	0.403
LH (IU/mL)	5.5 ± 2.6 (4.5–6.6)	5.1 ± 2.9 (4.4–5.8)	0.487
TSH (mIU/L)	2.10 ± 0.84 (1.78–2.43)	1.96 ± 0.84 (1.75–2.18)	0.467
BMI (kg/m^2^)	29.1 ± 1.4(28.5–29.6)	29.1 ± 1.5 (28.7–29.5)	0.853
Basal PRL level (ng/ml)	12.8 ± 6.5 (10.3–15.3)	14.1 ± 6.8 (12.4–15.8)	0.414
PRL before OPU (ng/ml)	15.7 ± 11.2 (11.4–20.1)	17.3 ± 11.8 (14.3–20.3)	0.551
PRL after OPU (ng/ml)	35.2 ± 25.4 (25.4–45.1)	41.4 ± 25.4 (35–47.8)	0.286
PRL before ET (ng/ml)	9.7 ± 7.6 (6.7–12.6)	12.3 ± 10.9 (9.5–15)	0.254
Number of oocytes retrieved in OPU	22.8 ± 9.6 (19.1–26.6)	14.8 ± 10.3 (12.2–17.4)	0.001^*^
Number of metaphase II oocytes	7.3 ± 2.6 (6.3–8.3)	5.1 ± 2.8 (4.4–5.8)	0.001^*^
Fertilization rate (%)	82 ± 13.1 (76.87–87.03)	81.4 ± 17.2 (77–85.7)	0.954
Number of embryos transferred	3.7 ± 0.7 (3.4–4)	3 ± 1.1 (2.7–3.2)	0.001^*^
Number of high quality embryos	3.5 ± 6.9 (3.1–3.8)	2.6 ± 1.3 (2.3–2.9)	0.002^*^
Number of low quality embryos	1.2 ± 0.4 (0.8–1.6)	1.1 ± 0.2 (0.9–1.2)	0.391

Values are given as mean ± SD (95% CI)

^*^Statistically significant result *p* < 0.05

### Relationship between PRL samples and other parameters

**Fig. 1:**
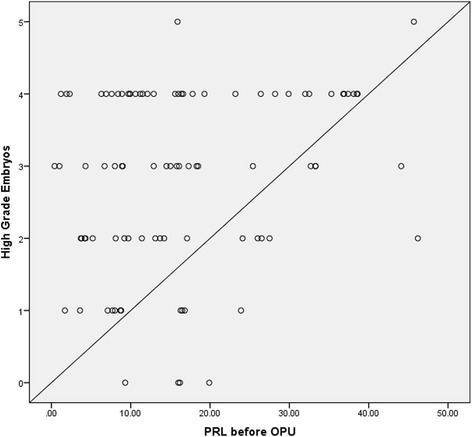
Scatter plot showing correlation between serum prolactin levels before OPU and high quality embryos

Table 4 shows a statistically significant positive pearson correlation between the prolactin level before OPU, and the number of higher quality embryos (*p* = 0.019). Higher quality embryos were defined as embryos with equal blastomeres, and fragmentation less than 30%. Figure 1 shows a scatter plot which illustrates the correlation between high quality embryos and serum PRL before OPU. There were no other correlations between the remaining prolactin samples, and higher, or lower quality embryos (unequal blastomeres, fragmentation ˃30%, multinucleation or other cytoplasmic defects).

**Table 4:** Correlation between serum prolactin levels, embryo quality & fertilization rate

	Higher quality embryos		Lower quality embryos		Number of retrieved oocytes		Fertilization rate	
	PC	*p*-value	PC	*p*-value	PC	*p*-value	PC	*p*-value
Basal PRL level	−0.182	0.085	0.108	0.607	−0.119	0.264	0.065	0.541
PRL before OPU	0.274	***0.019*** ^*^	0.245	0.237	−0.004	0.972	0.044	0.680
PRL after OPU	0.108	0.310	0.306	0.136	−0.135	0.205	−0.081	0.446
PRL before ET	−0.117	0.272	0.093	0.093	−0.218	***0.039*** ^*^	0.228	***0.030*** ^*^

^*^Statistically significant result *p* < 0.05

*PC* Pearson Correlation

There was also a statistically significant negative relationship between the number of oocytes retrieved, and serum prolactin level before embryo transfer as shown by pearson correlation. The same sample also showed a statistically significant positive correlation with fertilization rate. There were no other significant correlations between any other serum prolactin sample and fertilization rate, or the number of oocytes retrieved. The clinical application of this correlation is not of value, as chronologically the number of oocytes retrieved is already known, and the fertilization rate can be calculated before this sample can be drawn at a later time (before ET).

## Discussion

Serum prolactin levels vary in the body according to different stimuli, including sleep, pregnancy, stress, chest wall stimulation, food ingestion, and trauma. Stress has been shown to be a common cause for raising serum prolactin levels [1]. This may explain the significant rise in serum PRL after OPU. Many studies has documented the poor effect stress has on the success of IVF/ICSI [17–20]. Our study has clearly documented a state of transient hyperprolactinemia which was also documented by other authors [3, 4].

This rise in serum prolactin peaked after the ovum pickup procedure with some readings reaching a maximum recorded value of 93.2 ng/ml, but returning to normal levels before ET.

Boyers et al. [4] had similar results where the mean basal level of serum prolactin was comparable to ours. They had a mean increase in serum prolactin during OPU reaching values higher than we recorded. The level decreased after OPU but remained above the baseline. Their results also showed a return of serum prolactin to normal before embryo transfer.

Our results showed that elevated serum prolactin identified after OPU had no significant impact on pregnancy, as did the results from previous work by Doldi et al. [21] This was contradictory to the work presented by Zhong et al. [3] which stated that the rise in serum prolactin level ≥ 90 ng/ml dramatically affected pregnancy rate, by modulating the hypothalamo-pituitary ovarian axis, corpus luteum function and endometrial receptivity. Other studies [8–10] had findings similar to our own, confirming the presence of hyperprolactinemia without recording any deleterious effects on pregnancy.

In the study by Doldi et al. [21] they divided the patients into two groups and attempted to treat hyperprolactimenia in one group with cabergoline (Dostinex®; pharmacia and Upjohn; 500 μg per week), or bromocriptine (Parlodel®; Novartis; 2.5 mg per week), and placebo in the control group. The treatment started prior to ovarian stimulation, and continued till there was a decrease in plasma prolactin levels, and eventually they found no significant difference in the pregnancy rate.

We also found a positive correlation between higher quality embryo and the serum prolactin sample taken before OPU. This can theoretically help predict better embryo implantation and successful pregnancy, however the same sample failed to show statistical significance when it came to clinical pregnancy. Mendoza et al. [11] concluded that better quality oocytes and embryos resulted from follicles with higher steroid and prolactin levels.

Keeping in mind that the development of high quality embryos requires good quality oocytes, the presence of prolactin receptors or it mRNA has been detected in the matured oocyte, and its surrounding cumulus cells. Evidence also suggests that it participates in the development, and maturation of oocytes [22]. Hypoprolactinemia was reported to decrease cleavage and pregnancy rates [23]. Jinno et al. [24] studied the effect of giving bromocriptine in the cycle preceding IVF, and found that they had higher cleavage, fertilization, and pregnancy rates. They also reported a higher proportion of high quality embryos. This was attributed to better oocyte maturation which was achieved due to the rebound increase of prolactin after the discontinuation of bromocriptine, which restored post receptor responsiveness in granulosa cells. Moride et al. [25] also concluded that the bromcriptine rebound method yielded morphologically better embryos.

Zhong et al. [3] presented results stating that a higher prolactin level at the day of hCG trigger yielded more oocytes by showing a positive spearman correlation while failing to show prolactin in a multiple linear regression analysis equation, concluding that serum prolactin at the day of pick up cannot predict the number of oocytes retrieved. They also showed no marked difference in the fertilization rate. Our study showed no correlation between the serum prolactin samples drawn, and either fertilization rate or the number of oocytes retrieved in the 1st three samples.

However our study demonstrated in the sample taken before embryo transfer, a statistically significant positive pearson correlation with fertilization rate, and a negative relationship with the number of oocytes retrieved. Optimizing these results for clinical value is doubtful as the final serum prolactin sample is taken before embryo transfer, and so if any benefit is to come from their value, the sample has to predate the oocyte retrieval process or the fertilization procedure.

Some of the limitations to our study included not identifying a cut off value for PRL sample before OPU in predicting higher quality embryos. This finding was a secondary outcome, and couldn’t be predicted before the statistical analysis of the results. The inclusion criteria was not specific enough to exclude all causes of low cleavage rates, and poor embryo quality e.g. older age, low ovarian reserve, PCOS, and endometriosis, which may lead to bias, and low accuracy if receiver operating characteristic (ROC) analysis was done to determine a cut off value.

## Conclusions

In normoprolactinemic women a state of transient hyperprolactinemia is clearly identified in patients undergoing ICSI. Although its cause is not identified, it can be attributed to stress and anxiety. This increase appears to be harmless, and doesn’t affect the outcome of ICSI. A significant positive correlation was identified between higher quality embryos and prolactin before ovum pickup. More research is warranted to help increase the predictability of better quality embryos, and hence quantification of their implantation potential. This might help with embryo selection aiming at smaller number of embryos to be transferred per cycle, with a consequent decrease in the risk of multiple pregnancies without decreasing pregnancy rates.

## Abbreviations

AFC: Antral follicle count
AUC: Area under the curve
BMI: Body mass index
E2: Estradiol
ET: Embryo transfer
FSH: Follicle stimulating hormone
GnRH: Gonadotrophin-releasing hormone
hCG: human chorionic gonadotropin
HMG: Human menopausal gonadotropin
ICSI: Intracytoplasmic sperm injection
IVF: In vitro-fertilization
OPU: Ovum pick up procedure
PC: Pearson correlation
PCOS: Polycystic ovary syndrome
PRL: Prolactin
ROC: Receiver operating characteristic
β-HCG: beta chorionic gonadotrophin
